# P2Y12 blocker monotherapy after percutaneous coronary intervention

**DOI:** 10.1007/s12471-021-01582-7

**Published:** 2021-06-08

**Authors:** F. W. A. Verheugt, P. Damman, S. A. J. Damen, J. J. Wykrzykowska, E. C. I. Woelders, R. -J. M. van Geuns

**Affiliations:** 1 Department of Cardiology, Onze Lieve Vrouwe Gasthuis, Amsterdam, The Netherlands; 2 Department of Cardiology, Radboud University Medical Centre, Nijmegen, The Netherlands; 3 Department of Cardiology, University Medical Centre, Groningen, The Netherlands

**Keywords:** Aspirin, Clopidogrel, Ticagrelor, Prasugrel, Percutaneous coronary intervention

## Abstract

For secondary prevention of coronary artery disease (CAD) antiplatelet therapy is essential. For patients undergoing a percutaneous coronary intervention (PCI) temporary dual antiplatelet platelet therapy (DAPT: aspirin combined with a P2Y12 blocker) is mandatory, but leads to more bleeding than single antiplatelet therapy with aspirin. Therefore, to reduce bleeding after a PCI the duration of DAPT is usually kept as short as clinically acceptable; thereafter aspirin monotherapy is administered. Another option to reduce bleeding is to discontinue aspirin at the time of DAPT cessation and thereafter to administer P2Y12 blocker monotherapy. To date, five randomised trials have been published comparing DAPT with P2Y12 blocker monotherapy in 32,181 stented patients. Also two meta-analyses addressing this novel therapy have been presented. P2Y12 blocker monotherapy showed a 50–60% reduction in major bleeding when compared to DAPT without a significant increase in ischaemic outcomes, including stent thrombosis. This survey reviews the findings in the current literature concerning P2Y12 blocker monotherapy after PCI.

## Introduction

Besides lipid lowering strategies, antithrombotic therapy is the cornerstone of secondary prevention of coronary artery disease (CAD). By far the most commonly used agent is single antiplatelet therapy with aspirin [1]. For patients with an aspirin allergy or intolerance, antagonists of the platelet P2Y12 receptor are an alternative. For patients undergoing percutaneous coronary intervention (PCI) dual antiplatelet therapy (DAPT: aspirin combined with a P2Y12 blocker) has become the standard of care [2]. In patients who have undergone a PCI for acute coronary syndromes (ACS) 1 year of treatment is recommended [3, 4]. In elective patients 6 months of DAPT is preferred by most cardiologists, but prolonged treatment can be given with more complex anatomy or the use of multiple stents. Thus, all stented CAD patients are on DAPT for a period of time. The only serious side effect of DAPT is increased bleeding in comparison to aspirin alone.

This short review will address the safety and efficacy of a reduction in DAPT-related bleeding by discontinuing aspirin rather than the P2Y12 blocker at the time of DAPT cessation, as has recently been evaluated in large-scale randomised clinical trials.

## Pharmacology of platelet P2Y12 inhibition

**Fig. 1:**
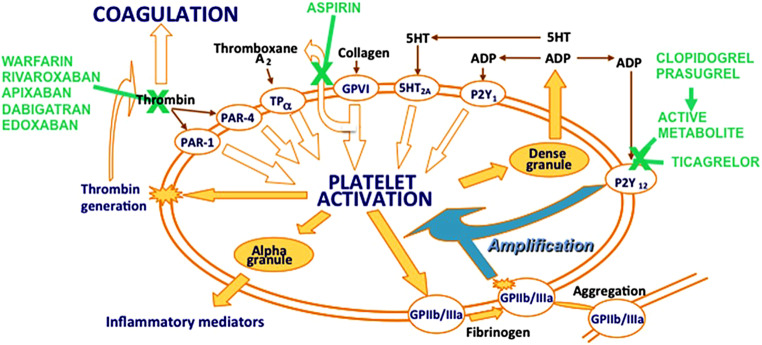
Pharmacology of antithrombotic agents in general, and of platelet P2Y12 inhibitors in particular. Reproduced from [5] with permission. *5‑HT* 5 hydroxytryptamine (serotonin), *ADP* adenosine diphosphate

Platelet release of thromboxane A2 and subsequent activation of platelets via the platelet P2Y12 receptor is an important pathway contributing to platelet aggregation and, consequently, thrombosis [5] (see Fig. 1). Activated platelets bind with their glycoprotein VI receptors to collagen after endothelial disruption, which further stimulates platelet thromboxane A2 release by inducing the cytosolic release of arachidonic acid, which is converted rapidly to thromboxane A2 via cyclo-oxygenase 1 (COX-1) and thromboxane synthase, even at very low doses. For example,, daily dosing with aspirin 25 mg leads to 95% inhibition of platelet thromboxane synthesis [6]. Standard once-daily dosing with aspirin 75–100 mg guarantees a consistent and very high level of COX‑1 inhibition in adherent patients [6].

In the platelets adenosine diphosphate (ADP) is stored at high concentrations in their dense granules and is released after platelet activation in response to thromboxane A2, thrombin and collagen, as well as ADP itself. ADP acting via P2Y12 receptors is not only one of the many platelet agonists to start platelet activation, but also amplifies platelet activation through P2Y12 receptors. These features have made platelet P2Y12 receptors a key target for preventing and treating arterial thrombosis [7].

The first P2Y12 inhibitor tested was ticlopidine, a prodrug that is metabolised in the liver to its active metabolite, but clinical application was terminated because of haematological side effects (agranulocytosis). Its successor drug is clopidogrel, which is also a thienopyridine prodrug that undergoes metabolism in the liver via cytochrome P450 (CYP) enzymes to generate the active metabolite that binds irreversibly to P2Y12 receptors to inhibit ADP binding. The pharmacodynamics of clopidogrel is limited in some individuals due to insufficient active metabolism, related to the loss-of-function variants of CYP2C19 and/or drug interactions and other factors making an individual response unpredictable even with genetic information [8].

Prasugrel is a thienopyridine prodrug, like clopidogrel, but is more efficiently transformed into its active metabolite without relevant effects of genetic variation in CYP activity or drug-drug interactions [9]. Therefore, it has a fast and more predictable platelet inhibition compared with clopidogrel and, thus, is more effective in the prevention of arterial thrombosis, and of stent thrombosis in particular.

Ticagrelor is also a platelet P2Y12 inhibitor that binds reversibly to the P2Y12 receptor and does not require metabolic activation for antiplatelet activity. Ticagrelor also has a more rapid and predictable platelet inhibition than clopidogrel [10]. Its therapeutic half-life is shorter than that of clopidogrel [11]. Ticagrelor has the most consistently high and less variable levels of platelet inhibition during long-term maintenance therapy compared with clopidogrel and prasugrel [12]. Ticagrelor 60 mg twice daily and 90 mg twice daily results in similarly high levels of platelet P2Y12 inhibition [13]. However, ticagrelor is often associated with the side effect of dyspnoea, which is usually mild, well-tolerated and transient; but sometimes dyspnoea may be intolerable and alternative therapy may be required [14].

## Clinical experience with P2Y12 blocker monotherapy in the pre-PCI era

Although aspirin was introduced in the 1980s as an antithrombotic agent in ACS, a randomised Italian trial in 652 patients with unstable angina evaluated ticlopidine 250 mg twice daily plus conventional therapy versus conventional therapy alone that did not include aspirin [15]. After 6 months the primary endpoint (vascular death and non-fatal myocardial infarction) was reduced by 46% from 13.6% to 7.3% (*p* = 0.009). Fatal and non-fatal myocardial infarction (including Q‑wave infarction) was reduced by 53% from 10.9% to 5.1% (*p* = 0.006). Mild bleeding was reported in only 4 patients, all in the ticlopidine arm. This medium-sized trial in ACS patients at risk for coronary artery (re)thrombosis shows the benefit of P2Y12 blocker monotherapy in the absence of aspirin. However, the study was performed in the pre-PCI era.

**Fig. 2:**
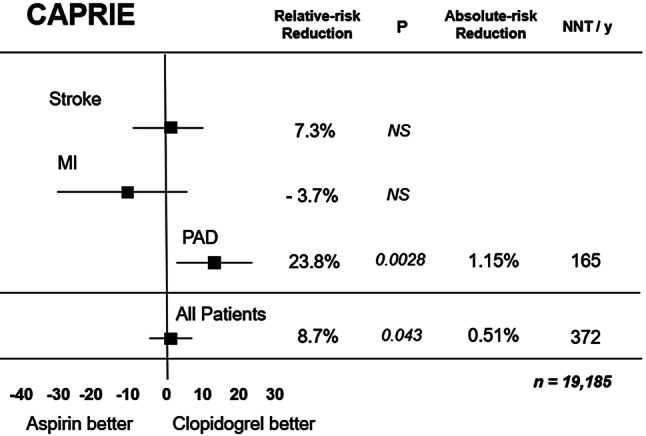
Vascular death, ischaemic stroke or myocardial infarction (*MI*) with clopidogrel versus aspirin in 19,185 vascular patients. After [16]. *MI* myocardial infarction, *NNT/y* number needed to treat per year, *PAD* peripheral artery disease

The largest clinical experience with P2Y12 blocker monotherapy was gained from the well-known CAPRIE trial published in 1996 [16]. Patients with stable vascular disease were randomised to clopidogrel 75 mg once daily without a loading dose, or to aspirin 325 mg once daily. The patients were stratified before randomisation for prior ischaemic stroke (*n* = 6431), peripheral vascular disease (*n* = 6452) or prior myocardial infarction (*n* = 6302). Patients in the latter group were eligible only if the myocardial infarction had occurred less than 30 days prior to study inclusion. After nearly 2 years the primary endpoint (vascular death, ischaemic stroke or myocardial infarction) was observed in 5.3% in the clopidogrel group and 5.8% on aspirin (*p* = 0.04), but this benefit was only seen in the peripheral artery disease subgroup and not in the patients with stroke or myocardial infarction at randomisation (Fig. 2). In the latter group recurrent myocardial infarction occurred in 2.8% on clopidogrel and in 2.8% in the aspirin group (*p* =NS), where there was no difference in vascular death or ischaemic stroke either. Although there was no significant difference in the total number of severe bleeding events or intracranial haemorrhages, major gastrointestinal bleeding was less frequent with clopidogrel (0.5%) than with aspirin (0.7%, *p* = 0.05). Thus, the CAPRIE trial showed that in patients with recent myocardial infarction clopidogrel monotherapy seems to be non-inferior to aspirin monotherapy, but appears to be safer. Whether this is also true for the currently used low dose of aspirin (75–100 mg daily) is uncertain. Moreover, the CAPRIE trial was performed in the pre-PCI era.

## P2Y12 blockers in PCI

Today, PCI is almost always performed with coronary stenting. To prevent thrombosis of the implanted stent(s), DAPT with a P2Y12 antagonist plus aspirin is absolutely indicated. Oral anticoagulation proved to be inferior to DAPT [17]. In the beginning ticlopidine was part of the DAPT regimen after coronary stenting, but the drug proved to be toxic (see above). Clopidogrel showed similar efficacy [18] and remains the standard P2Y12 antagonist in elective coronary stenting, whereas ticagrelor and prasugrel are now mandatory after stenting in ACS [3, 4].

It became clear almost immediately that DAPT causes excess major bleeding in comparison to aspirin alone in the first month after stenting [19], including fatal bleeding [20]. Also long term DAPT is associated with more major bleeding than occurs with aspirin alone [21]. To minimise the risk of bleeding after PCI careful balancing between thrombotic risk and bleeding risk is essential. For that purpose two established risk scores have been developed: the DAPT [22] and PRECISE-DAPT [23] scores. These help clinicians to estimate the optimal duration of DAPT.

While at the end of the optimal duration of DAPT the P2Y12 blockers are usually stopped and aspirin is continued, an intriguing option has recently been put forward: continuation of P2Y12 blocker therapy and discontinuation of aspirin. In other words: is P2Y12 blocker monotherapy after DAPT cessation effective and safe?

## Recent trials with P2Y12 blocker monotherapy after PCI

Between 2018 and 2020 five aspirin-controlled randomised clinical trials of P2Y12 blocker monotherapy after DAPT cessation in 32,181 stented patients were published [24–28]. Except for the TICO trial [28] they included both ACS and stable patients. All studies were multicentre and open label, except for TWILIGHT, which was double blind for aspirin or placebo. Bleeding was the primary endpoint in only two studies, ischaemic endpoints in two, and combined bleeding and ischaemic events in one trial. Two meta-analyses on these P2Y12 blocker monotherapy studies have been published recently: one is a systemic review of the five trials above [29] and one is a network meta-analysis on DAPT duration after drug-eluting stent (DES) implantation in general [30]. Unfortunately, the latter did not contain the TICO trial on P2Y12 antagonist monotherapy after ACS [28], which has been included in the current survey. The results of the five trials are summarised in Tab. 1 and 2.

**Table 1:** Bleeding and ischaemic events in the five randomised aspirin-controlled trials on P2Y12 blocker monotherapy after percutaneous coronary intervention

Trial	*n*	ACS %	Elective %	DAPT	P2Y12 monotherapy	Bleeding		Ischaemic endpoints	
						Experimental	Control	Experimental	Control
*GlobalLeaders *[24]	15,996	47	53	1 month	Ticagrelor 23 months	2.1%^a^	2.1%^a^	3.8%^b^	4.3%^b^
*SMARTCHOICE* [25]	2,993	58	42	3 months	Clopidogrel^c^ 9 months	2.0%^d^	3.4%^d^	2.9%^e^	2.5%^e^
*STOPDAPT2 *[26]	3,045	38	62	1 month	Clopidogrel 11 months	0.5%^f^	1.8%^f^	2.4%^g^	3.7%^g^
*TWILIGHT *[27]	7,119	65	35	3 months	Ticagrelor 12 months	4.0%^h^	7.1%^h^	3.9%^i^	3.9%^i^
*TICO *[28]	3,056	100	0	3 months	Ticagrelor 9 months	3.9%^j^	5.9%^j^	1.7%^k^	3.0%^k^

^a^ Any bleeding

^b^ Total mortality or new Q‑wave myocardial infarction (MI) (primary endpoint), *p* = 0.07

^c^ Ticagrelor or prasugrel in acute coronary syndrome patients (around 55%)

^d^ BARC type 2–5, *p* = 0.02

^e^ All-cause mortality, MI or stroke (primary endpoint), *p* = 0.007

^f^ BARC type 3 or 5, *p* = 0.003

^g^ Cardiovascular death, MI, definite stent thrombosis, stroke or TIMI major or minor bleeding (primary endpoint), *p* = 0.04, *p* < 0.001

^h^ BARC type 2, 3 or‑5 (primary endpoint), *p* < 0.001

^i^ All-cause mortality, non-fatal MI or non-fatal stroke

^j^ TIMI major bleeding, *p* = 0.02

^k^ Major bleeding and major adverse cardiac and cerebrovascular events (primary endpoint), *p* = 0.01

**Table 2:** Bleeding and ischaemic events in four randomised aspirin-controlled trials on P2Y12 blocker monotherapy after percutaneous coronary intervention in acute coronary syndrome patients (after [29])

Trial	*n*	Bleeding HR (95% CI)	Ischaemic endpoints HR (95% CI)
*GlobalLeaders* [24]	7,487	0.52 (0.33–0.81)	0.73 (0.51–1.03)
*SMARTCHOICE* [25]	1,741	0.56 (0.33–1.05)	1.06 (0.61–1.85)
*TWILIGHT* [27]	4,614	0.47 (0.36–0.61)	0.97 (0.73–1.28)
*TICO* [28]	3,056	0.56 (0.34–0.91)	0.69 (0.45–1.06)
*Total*	*16,898*	*0.50 (0.41–0.61)*	*0.85 (0.70–1.03)*

*HR* hazard ratio, *CI* confidence interval

Bleeding was nearly halved by P2Y12 blocker monotherapy compared to DAPT, underscoring the large contribution of aspirin to bleeding in DAPT. Discontinuation of aspirin did not lead to an excess of major adverse cardiovascular events (MACE). In particular, myocardial infarction was not affected by discontinuing aspirin after DAPT cessation, nor was stent thrombosis, which occurred in less than 0.5% of the patients. In general, these meta-analyses show that P2Y12 monotherapy is associated with less major bleeding and a similar incidence of stent thrombosis, all-cause mortality, myocardial infarction and stroke compared with prolonged DAPT. These results were seen in both stable patients as well as in those stented for ACS (Tab. 2).

## Implications of P2Y12 blocker monotherapy after PCI

The finding that P2Y12 blocker monotherapy after aspirin cessation reduces major bleeding without increasing long-term ischaemic endpoints has not yet been incorporated in the current guidelines. All five trials used different endpoint definitions especially for ischaemic events, which makes comparability difficult. Moreover, only one trial was double blind for aspirin. DAPT durations were also different, as were long-term follow-up periods. Although the number of patients in the trials exceeded 32,000, to prove non-inferiority as regards ischaemic outcome more trials are needed, especially because the current rate of stent thrombosis is lower than 1%. Furthermore, three of the five trials were carried out in eastern Asia, where patients are poor metabolisers of clopidogrel, the Achilles heel of clopidogrel. Apparently, however, this did not lead to ischaemic problems. Yet, before such a strategy with clopidogrel monotherapy is applied widely, genotyping of the patients seems essential [31]. This test is simple and widely available. After PCI, carriers of CYP2C19*2 or CYP2C19*3 loss-of-function alleles should not be treated with clopidogrel monotherapy and should be continued on aspirin, or switched to ticagrelor monotherapy. Unfortunately, the latter option may have financial consequences in many parts of the world, potentially leading to poor compliance. ACS patients treated with DAPT can be continued on ticagrelor monotherapy at 3 months after PCI, as shown in the TWILIGHT trial [27]. Also the antithrombotic potency of ticagrelor monotherapy proved similar to that of ticagrelor plus aspirin with respect to ex vivo blood thrombogenicity [32].

Whether the above results also apply for prasugrel is uncertain. A small uncontrolled safety study (ASET) evaluated prasugrel monotherapy in 202 stable patients at low ischaemic risk. They were uploaded with DAPT (aspirin and clopidogrel) prior to PCI. If the angiographic result was satisfactory, patients were uploaded with 60 mg of prasugrel, after which prasugrel 10 mg was continued for 3 months without aspirin [33]. The primary endpoint was stent thrombosis, which was not seen in any patient. There was one fatal intracranial haemorrhage 6 h after PCI. It is unknown whether a large and aspirin-controlled study with prasugrel monotherapy will follow.

A group of patients that may also benefit from P2Y12 blocker monotherapy are those with aspirin allergy. Most guidelines recommend clopidogrel as an alternative to aspirin, but poor metabolism of clopidogrel represents a problem. Here again, ticagrelor may be the better option, since the GlobalLeaders study showed ticagrelor monotherapy not to be inferior to aspirin monotherapy [24].

Whether patients on ticagrelor monotherapy benefit more than those on clopidogrel monotherapy or prasugrel monotherapy cannot be analysed here, because each of the five trials used either ticagrelor monotherapy or clopidogrel monotherapy.

Finally, there was no clear difference in outcomes in patients with either 1 or 3 months of DAPT before P2Y12 blocker monotherapy.

## Bleeding while on DAPT and P2Y12 blocker monotherapy as an option

If patients bleed while on DAPT post-PCI physicians usually stop the P2Y12 blocker and continue aspirin, but this algorithm may change. When bleeding occurs during DAPT, in most cases the stronger P2Y12 blockers (ticagrelor and prasugrel) are changed to clopidogrel with its inherent ischaemic risks. Excess bleeding with the stronger PY12 blockers is not only seen in the registration trials, but also in large registries in Sweden [34] and the Netherlands [35]. Rather than stopping the P2Y12 blocker, discontinuation of aspirin may be an attractive alternative given the safety results of the five trials on P2Y12 blocker monotherapy [29]. The TWILIGHT trial in particular clearly showed that the contribution of aspirin to bleeding during DAPT is rather large: the risk of bleeding while on DAPT is halved after discontinuation of aspirin at 3 months [27].

## Future perspectives on P2Y12 blocker monotherapy

**Fig. 3:**
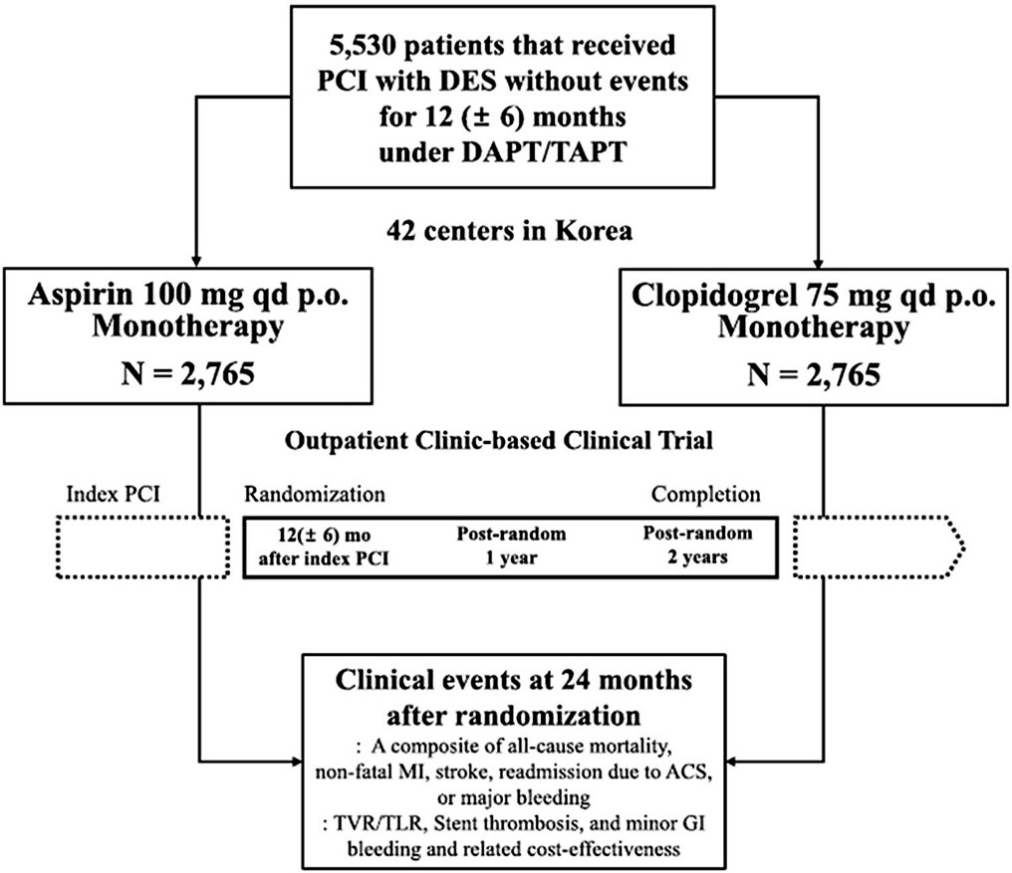
Design of the currently running HOST-EXAM trial in Korea (NCT02044250). Reproduced from [36] with permission. *ACS* acute coronary syndrome, *DAPT* dual antiplatelet platelet therapy, *DES* drug-eluting stent, *GI* gastrointestinal, *MI* myocardial infarction, *PCI* percutaneous coronary intervention, *TAPT* triple antiplatelet therapy, *TLR* target lesion revascularisation, *TVR* target vessel revascularisation

Although P2Y12 blocker monotherapy after PCI seems to be successful in five well-performed published trials with more than 32,000 patients followed for at least 12 months, more data are necessary before this strategy will be widely adopted. The generalisability of P2Y12 blocker monotherapy will be enhanced when more trials and registries have been published. In particular, data on very long-term outcomes of P2Y12 blocker monotherapy are lacking. To the best of our knowledge, there is one pertinent trial that is currently being carried out in Korea in nearly 6000 PCI patients: the HOST-EXAM (NCT02044250) study [36]. In that trial, patients who have undergone PCI and have been treated with DAPT without events are randomised at the time of DAPT cessation (12–18 months post-PCI) to long-term clopidogrel monotherapy or long-term aspirin monotherapy (Fig. 3). Presentation of the results is expected in mid-2021.

## Conclusion

To prevent bleeding in patients on DAPT post-PCI an interesting and intriguing option is P2Y12 blocker monotherapy after DAPT cessation. In several recent aspirin-controlled trials P2Y12 blocker monotherapy halved the bleeding risk when aspirin was discontinued at the time of DAPT cessation and did not seem to increase the number of ischaemic events such as myocardial infarction or stent thrombosis. These results were seen in both stable patients as well as in those stented for ACS. New guidelines will address this new, relatively simple topic. The cardiology community will possibly widely adopt long-term P2Y12 blocker monotherapy, as neurologists have done since the 1996 CAPRIE study [16].
